# The rate of ileostomy site incisional hernias: more common than we think?

**DOI:** 10.1007/s10029-024-03163-0

**Published:** 2024-09-26

**Authors:** Megan Obi, Lucas Beffa, Megan Melland-Smith, Nir Messer, Arielle Kanters, Sami Judeeba, Kevin Baier, Benjamin Miller, David Krpata, Ajita Prabhu, Scott R. Steele, Michael Rosen, Stefan D. Holubar, Clayton Petro

**Affiliations:** 1 Department of General Surgery, Cleveland Clinic Foundation, Cleveland, OH USA; 2 Center for Abdominal Core Health, Digestive Disease Institute, Cleveland Clinic Foundation, 9500 Euclid Ave, Cleveland, OH 44195 USA; 3 Department of Colorectal Surgery, Cleveland Clinic Foundation, Cleveland, OH USA

**Keywords:** Diverting loop ileostomy, Ostomy, Stoma, Incisional hernia, Stoma reversal

## Abstract

**Purpose:**

Incisional hernias (IH) rates after diverting loop ileostomy reversal (DLI-R) have been reported up to 24%. We aimed to characterize the incidence rate and risk factors associated with DLI-R site IH formation within 1-year in a large patient cohort.

**Methods:**

A retrospective review at a single quaternary referral center hospital of adult patients who underwent DLI-R over a 5-year period and abdominal computerized tomography (CT) imaging performed within 1-year for any indication postoperatively was conducted. All CTs scans were independently reviewed by staff surgeons to determine the presence of a fascial defect at the DLI-R site.

**Results:**

2,196 patients underwent DLI-R; of these, 569 (25.9%) underwent CT imaging for any indication. Mean patient age, 54.8 (± 14.9), BMI 27.6 kg/m^2^. 87 (15%) patients had a parastomal hernia at time of DLI-R. After median follow-up of 10 months, 203 patients (35.7%) had IH at the DLI-R site. Age (*p* = 0.14), sex (*p* = 0.39), race (*p* = 0.75), and smoking status (*p* = 0.82) weren’t associated with IH after DLI-R. Comorbidities weren’t significantly associated with IH following DLI-R. In univariate analysis, increased BMI (*p* < 0.001), presence of a parastomal hernia (*p* = 0.008), and suture type (*p* = 0.01) were associated with IH development. On multivariate analysis, BMI remained significant, and polyglyconate compared to polydioxanone suture were associated with higher rates of IH (*p* < 0.001).

**Conclusion:**

We observed that the rate of incisional hernias within 1-year of diverting ileostomy reversal was indeed common at 36%. Granted, a high percentage of the population was excluded due to heterogeneity in radiographic evaluation that could be mitigated in future prospective studies. Our study suggests that IH preventative strategies include weight loss for overweight and obese patients prior to DLI-R and that the optimal suture for DLI-R is polydioxanone.

**Supplementary Information:**

The online version contains supplementary material available at 10.1007/s10029-024-03163-0.

## Introduction

Diverting loop ileostomies (DLIs) are commonly utilized adjunct procedures for the management of both benign and malignant colorectal diseases. They are utilized to protect high-risk anastomoses and to reduce the severity of leaks when they occur [1]. Over 100,000 new ostomies are created annually, and while there are several benefits to stoma creation, they are also associated with significant morbidity and decreased quality of life [1, 2]. Prior studies have reported DLI morbidity rates of up to 50% of cases with complications such as high stoma output, skin changes including dermatitis and ulceration, and stoma prolapse, obstruction, and herniation commonly identified [1, 3, 4]. Given the high rate of morbidity, optimal timing for stoma closure is important to determine for each patient.

Approximately 60–80% of all DLIs undergo reversal (DLI-R) [5]. While reversal can alleviate some of the negative impacts on renal function and quality of life, reversal also comes with its own risks. There are several known complications related to stoma reversal, including anastomotic leakage (1%), obstruction (14%), wound infection (9%), and incisional hernia (IH) [6, 7]. IHs have been reported in 7–35% of post- stoma reversals [8, 9]. Of those reported, prior studies have found that approximately 50% of those will require surgical repair which is a procedure which comes with its own significant risks [10]. Prophylactic management at the time of reversal has been considered although is not widely utilized due to concerns regarding mesh related complications as well as lack of high-volume studies [11].

This study aimed to better characterize the rate of ileostomy site incisional hernias (ISIH) after DLI reversal to further understand if additional measures should be considered at the time of reversal. To date, studies have evaluated IH rates in both ileostomies and colostomies in their analysis or have been limited to DLIs for rectal cancer if they excluded colostomies (Table 1) [8, 9, 12–16]. Therefore, we aimed to characterize the rate and risk factors associated with ISIH formation within 1-year after DLI-R in a high-volume colorectal surgery practice. We hypothesized that ISIH rates are more common than those previously reported.

## Methods

This retrospective review evaluated the frequency of radiographic ISIH ileostomy sites via medical record review and evaluation of computed tomography (CT) scans. The Institutional Review Board at Cleveland Clinic reviewed this study and found it exempt. Written informed consent from patients was not required as this was a retrospective study. All patients > 18 years of age who had a DLI-R between January 1, 2017, and December 31, 2022, at the Department of Colorectal Surgery, Cleveland Clinic Foundation, were identified by natural language processing of CPT codes in the hospital medical records. All DLI-R operations were performed by members of our local and regional colorectal surgery teams. Regular follow-up of all patients was performed within the same institution, but follow-up schedule was heterogenous as indication for DLI and subsequent reversal ranged from benign to malignant. Cases in which mesh reinforcement was noted at the time of DLI reversal or in which the decision was made to place the mesh during the DLI-R were excluded.

**Fig. 1:**
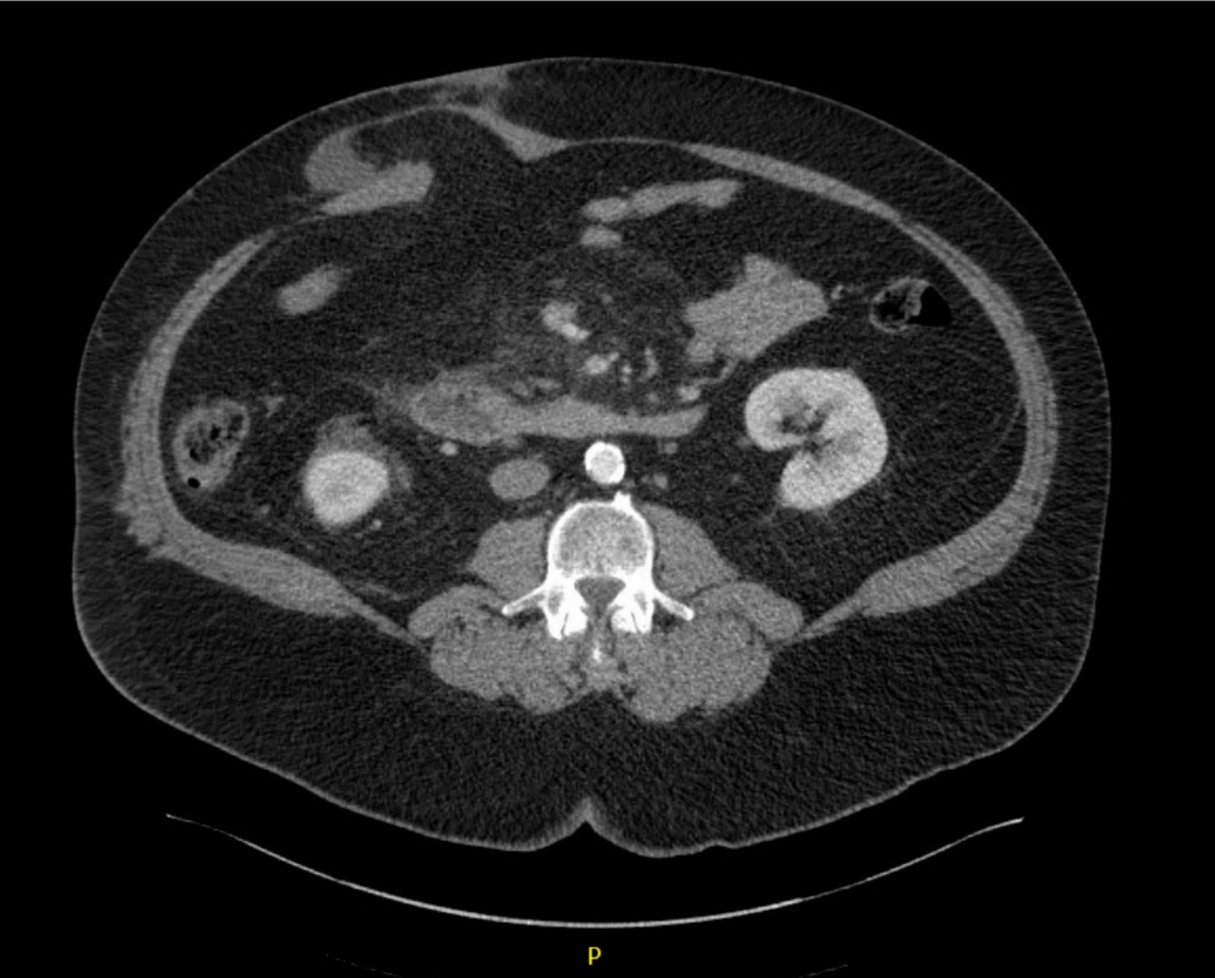
Ileostomy Site Incisional Hernia

Radiographic images were individually reviewed by members of the colorectal and abdominal wall reconstruction teams (LB, MMS, NM, AK, SJ, KB, BM, DK, AP, SRS, MR, SDH, and CP) for presence of ISIH. The definition of ISIH was “Any abdominal wall gap with or without [a] bulge in the area of a postoperative scar perceptible or palpable by clinical examination or imaging” (as described by Korenkov *et al.)* for which our surgeons felt they would offer surgical repair [17]. (Fig. 1)

Data collected from the electronic medical records included patient demographics, operative data, radiographic data, and medical history. Demographic data included age, sex, race, body mass index (BMI), and ethnicity. Operative data included case length, length of stay (LOS), presence of parastomal hernia noted by the surgeon on the operative report, estimated blood loss (EBL), and ASA rating. The method of closure of the stoma site, including the suture type used for closure, technique for closing the fascia, and surgical approach, was assessed in all cases. Operative technique was left to surgeon discretion. Radiographic data included the date of CT and CT type. CT scans for any indication (i.e., symptomatic hernia, evaluation of alternative disease processes, routine evaluation) were included in the analysis and needed to be performed within 1 year ± 6 months from the date of DLI-R. Medical history included a history of diabetes (DM), myocardial infarction (MI), coronary artery disease (CAD), congestive heart failure (CHF), stroke or transient ischemic attack (TIA), peripheral vascular disease (PVD), chronic obstructive pulmonary disease (COPD), liver disease, chronic kidney disease (CKD), and smoking history. Indications for initial DLI included but were not limited to the management of rectal cancer, inflammatory bowel disease (IBD), diverticulitis, dysmotility, colon cancer, and polyposis. The primary endpoint was the rate of ISIH as defined by the presence of hernia on CT, as CT is the optimal method for diagnosing incisional hernia [18]. Univariate and multivariate risk factor analyses for the development of ISIH were also performed.

### Statistical analysis

Continuous variables are presented as mean (SD), median (25th, 75th ), or frequency (proportion). Categorical variables are presented as frequency (proportion). Continuous variables were tested using Welch’s two-sample *t*-test to determine differences between those who developed ISIH and those who did not. Univariate analysis for categorical variables was performed using Pearson’s chi-square test. A multivariable logistic regression model was created to adjust for the following variables: BMI, diabetes status, presence of parastomal hernia, and suture material. The remaining variables were not included in the multivariable model due to a lack of significant association with the outcome of interest in the univariate analysis. A significance level of 0.05 was used. Data were analyzed using R, version 4.2.2 (RStudio, Boston MA).

## Results

**Fig. 2:**
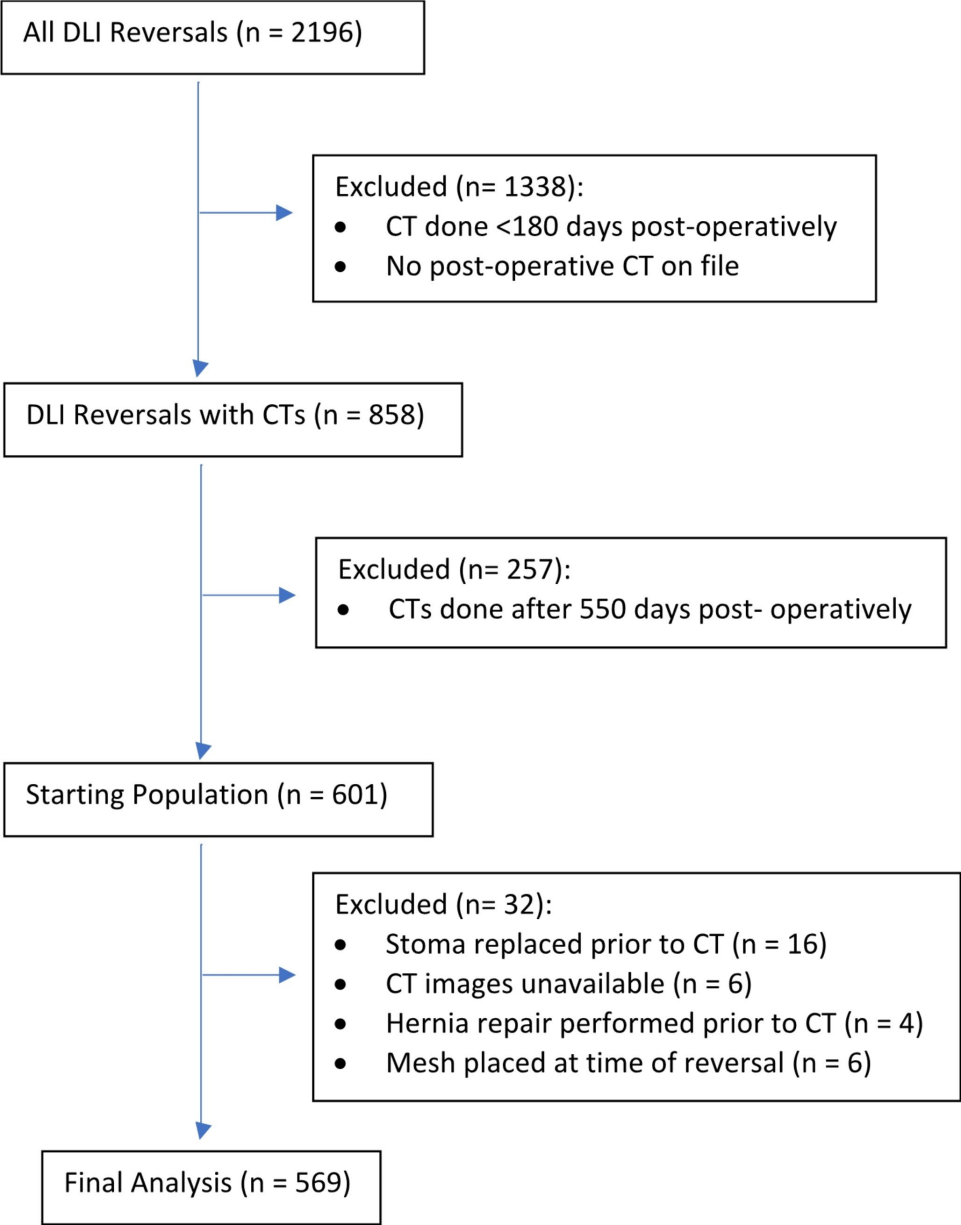
Consort Diagram

Initial data collection returned 2,196 records, of which 1,338 (60.9%) were excluded owing to a lack of 6-month postoperative CT imaging availability. The subsequent 858 (39.1%) records were re-reviewed, and an additional 257 (30.0%) records were excluded because CT imaging was performed 550 days postoperatively. The remaining 601 records had their radiographic imaging reviewed, and an additional 32 records were excluded due to lack of accessible CT imaging, hernia repair performed during the study period, mesh noted or placed at time of reversal, or stoma replaced during the study period. A total of 569 (25.9%) patients were identified as having previously had a DLI that underwent DLI-R in addition to having a CT scan performed within the appropriate study window (Fig. 2). Median follow-up for CT imaging was 10 (range 6–18) months. The cumulative rate of CT-verified ISIH was 35.7% (*n* = 203).

### Baseline characteristics

Of the 569 patients, 289 (50.8%) were female and 280 (49.2%) were male, with a mean age of 55-years-old. Most patients were white (84%) or non-Hispanic (96.3%). Fifty-nine patients (10.4%) were identified as black and 16 (2.8%) as Hispanics. The median BMI was 27.6 kg/m^2^ (13–53) with 32.5% qualifying as obese (BMI ≥ 30) and 2.5% as underweight (BMI ≤ 18). Most patients had a current (11.8%) or past (41.7%) smoking history. The comorbidities and risk factors included diabetes (18.5%), CAD (12.1%), CKD (10%), COPD (9.8%), CHF (7.2%), TIA/stroke (5.3%), PVD (4.2%), MI (4.2%), and liver disease (3.0%). Prior to DLI-R, parastomal hernias were observed in 87 (15.3%) of patients (Table 2).

### Demographics and risk factors

On univariate analysis, there were no significant differences in age (*p* = 0.14), gender (*p* = 0.39), race (*p* = 0.75), and smoking status (*p* = 0.82) of those who developed ISIH after DLI-R and those who did not. An increased BMI (30.4 vs. 26.1 kg/m^2^) was identified as a risk factor (*p* < 0.001) for the development of ISIH. There were no significant differences in comorbidities (DM, MI, CAD, CHF, TIA/stroke, COPD, CKD, PVD, or liver disease) between patients who developed ISIH and those who did not. Although while the presence of each individual co-morbidity was insignificant, the presence of any combination of co-morbidities was found to be significant (*p* = 0.04; Table 2).

### Operative risk factors

After DLI-R, there was no significant difference in the LOS (*p* = 0.92) and case length (*p* = 0.91) between those who did and did not develop ISIHs. However, those who were noted to have a parastomal hernia at the time of DLI reversal were significantly more likely to develop ISIH (21% vs. 12%; *p* = 0.008). Most cases (93.0%) were performed via the trephine, and there was no significant difference in ISIH in the operative technique (*p* = 0.85), whether via the trephine (93.6% vs. 92.7%), laparotomy (4.4% vs. 4.6%), or laparoscopy (2% vs. 2.7%). Polydioxanone (PDS^®^) was the most commonly utilized suture (56.8%) for fascial closure, and there was a significant difference in the ISIH rate among suture materials (*p* = 0.01), while the fascial closure technique (running or interrupted fashion) was not significant (*p* = 0.37). The ISIH rates when 0, 1, 2, 3, or all four significant risk factors (BMI, parastomal hernia presence, history of any co-morbidity, and suture material type) were present on univariate analysis (Table 3) were 16%, 35.7%, 33%, and 2.6%, respectively (*p* < 0.001).

### Multivariate analysis of risk factors for ISIH

Multivariable logistic regression analysis of risk factors associated with ISIH demonstrated that overweight (BMI 25–30; *p* < 0.001) and obese (BMI ≥ 30; *p* < 0.001) patients had 3x and 7x, respectively, increased odd of developing ISIH, respectively, compared to normal BMI patients. Presence of any co-morbidity (*p* = 0.13) and a parastomal hernia at the time of DLI-R (*p* = 0.6) had no significant impact on the development of ISIH. Compared with the use of polydioxanone for fascial closure, polyglyconate (Maxon™) had 2x increased odd of ISIH development (*p* < 0.001). Polypropylene (Prolene^®^) and polyglactin (Vicryl^®^) had non-significant (*p* = 0.13 and *p* = 0.8) decreased odds of ISIH compared with polydioxanone (PDS^®^). The ISIH rates when 0, 1, or 2 of these significant risk factors were present on multivariate analysis (Table 4) were 24.3%, 55.7%, and 20%, respectively (*p* < 0.001).

## Discussion

The radiographic rate of ISIH following DLI closure may be higher than previously reported. In this study, approximately 36% of patients who underwent DLI reversal subsequently developed a radiographic incisional hernia within 1 year of surgery. In addition, the BMI and suture type used for stoma fascial closure may be associated with subsequent IH development. Thus, our study suggests that preventive strategies include weight loss in overweight and obese patients and that the optimal suture is polydioxanone (PDS^®^).

Prior literature has previously demonstrated that high levels of IH hernias after stoma reversal but have been heterogeneous in their inclusion criteria, have not all been representative of the American population, have had low sample sizes, and their rates have not reached the level noted in this study (Table 1) [9, 10, 12, 13, 16, 19–21]. The vast majority of IH after stoma repair literature includes both end colostomies (ECs) as well as DLIs. IH rates from these studies ranged from 15.7 to 34.6%, lower than observed in our study [9, 12]. The inclusion of ECs in these studies could be diluting the observed rate of IHs from DLIs, but as a tertiary referral center referral bias may have influenced our ISIH rate. Studies that only included DLIs were mostly specific to rectal cancer management and reported rates ranging from 7.4 to 14.9% [8, 14]; these rates are likely an under-representation of the incidence of IHs. In addition, most studies involved international patients, which is significant given the differences in the baseline characteristics. BMI among Americans is significantly different, and as was noted in this study as well as prior studies, BMI can significantly impact the development of IHs [9, 12, 14, 20–26].

This study benefits from focusing exclusively on IH rates after all indications for DLI, involves an American patient population, and has the highest sample size studied to date. While our rate is higher than that of previously published data, there are also several factors that are important to contextualize the difference. The method by which a hernia was defined in this study compared to prior studies may differ, and this study focuses only on patients with subsequent radiographic imaging; thus, those without may alter the final rate. Overall, these data imply that the high selectivity of prior studies may have underrepresented the observed IH rate after DLI reversal.

As with all hernia studies, understanding the risk factors associated with hernia development can significantly impact pre-, intra-, and postoperative decision-making. In prior studies, BMI was the most cited positive risk factor for IHs post-stoma reversal, and this trend continued in this study [8, 9, 12, 14, 20, 21, 25–27]. This finding is unsurprising, as obesity has frequently been associated with IH development and recurrence [28, 29]. Unlike our study, co-morbidities (i.e. HTN, DM, and heart disease), the presence of parastomal hernia, history of surgical site infections, and sex have been previously associated risk factors for IH. Previous studies have had smaller sample sizes; thus, the impact of these factors may have initially appeared more prominent.

Regarding suture type, this study found non-significant differences between absorbable and non-absorbable sutures. This is in line with prior studies that also found no notable difference between the two [9, 20]. This study highlighted a possible difference in effectiveness between polyglyconate and polydioxanone, both absorbable monofilament sutures, which are characterized by differences in their handling, memory, and tensile strength [30]. Utilization of either material is usually institution-dependent based on availability, but this study suggests that, while overall similar, their indications may differ and thus their utilization might not be interchangeable.

Approximately 50% of all IHs that are acquired post-DLI reversal will require surgical repair, and hernia repair is a known high-risk procedure with notable morbidity and mortality [10, 31, 32]. Prophylactic measures have been considered as an option to reduce the subsequent need for future IH surgical repair; however, the level of efficacy remains low, and thus, the intervention is minimally utilized [33].

Several retrospective and prospective studies have investigated this topic. Warren et al. performed a retrospective review of all ostomy reversals that utilized synthetic mesh reinforcement and found a significant decrease in surgical site infections and stoma site hernias [13]. The ROCSS study is an early randomized control trial that assessed whether the utilization of biological mesh could reduce the incidence of IHs at stoma closure sites. They noted that there was a significant reduction in radiographic hernia rates as well as a non-significant decrease in symptomatic hernias and need for surgical intervention in those who received mesh vs. suture closure without any notable complications [34].

In a smaller single-center prospective study, the SCAR trial, the utilization of a macroporous polypropylene mesh placed in the retrorectus position during DLI closure was found to be associated with minimal post-operative complications and a low number of IHs in the short-term [35]. This study does not assess prophylactic techniques, but with the high rate of post-operative incisional hernias and the relative success demonstrated in prior studies, it does beg the questions whether there is more we can and should be doing when performing DLI closures. When discussing options for augmented mesh or mesh suture fascial closure, patients and surgeons need to have a true understanding of the risk of traditional fascial closure to make an educated decision about whether additional adjuncts are warranted.

Our study has a number limitations. The first is the retrospective nature of this study, which lends itself to selection bias in that we were unable to account for patients who were lost to follow-up and the inconsistency in which patients ultimately underwent radiographic evaluation (Supplementary Table 1). While our institution regularly treats a diverse population of local, national, and international patients, this study was a single-institution review from a tertiary center and may not be generalizable. In addition, this study only assessed the radiographic hernia rate, and did not consider which hernias were symptomatic and ultimately required surgical intervention. While prior studies have found that the positive predictive value of symptoms (i.e., bulge, pain) was low, from a patient standpoint understanding the symptomatic rate could still be of value [8]. This study was also unable to assess the hernia rates in those who did not ultimately have any reason to get a radiographic scan during the study period and thus could significantly affect the final determined rate.

## Conclusions

The rate of incisional hernia after diverting loop ileostomy closure may be higher than previously reported. In this study, approximately one in three patients who underwent DLI reversal developed a radiographic incisional hernia despite the fact that only 1 in 4 patients were evaluated due to heterogeneity in radiographic evaluation post-reversal. There may be an association between the type of suture material used and subsequent IH development. Prophylactic techniques may be worth considering for highly selective patient populations. Our study suggests that preventative strategies include preoperative weight loss for overweight and obese patients, and that the optimal suture is polydioxanone (PDS^®^). Given the high incidence of ISIH after DLI-R, further studies of prophylactic hernia repair at the time of DLI-R in these high-risk patients are warranted.

**Table 1:** Prior retrospective studies of hernia rates after stoma reversal

First Author	Year	Country	*N*	Hernia Rate	Included Stomas	Risk Factors
Guzman (Guzmán-Valdivia, 2008)	2008	Mexico	70	31.4%	DLIs and Colostomies	COPD, ischemic heart disease, DM
Schreinemacher (Schreinemacher et al., 2011)	2011	Netherlands	111	32.4% (ultrasound)	DLIs and Colostomies	BMI
Bhangu (Bhangu, Fletcher, et al., 2012)	2012	England	59	34% 31% radiographic	DLIs and Colostomies	n/a
Sharp (Sharp et al., 2015)	2015	USA	365	19%	DLIs and Colostomies	Age, DM, colostomies, BMI, urgent operation
De Keersmaecker (De Keersmaecker et al., 2016)	2016	Belgium	153	11.1%	DLIs after rectal cancer	None
Fazekas (Balazs et al., 2017)	2016	England	121	14.9%	DLIs after LAR for rectal cancer	BMI, open surgery, prior hernia, longer time to reversal
Oriel (Oriel et al., 2017)	2017	USA	114	9.6%	DLIs & Colostomies	SSI
Amelung (Amelung et al., 2018)	2018	Netherlands	318	34.6%	DLIs & Colostomies	BMI, stoma prolapse, parastomal hernia, HTN
Brook (Brook et al., 2018)	2018	Ireland	139	13.5%	DLIs	SSI, BMI
Kaneko (Kaneko et al., 2019)	2018	Japan	134	23.9%	DLIs	BMI, HTN, prior IH
Warren (Warren et al., 2018)	2018	USA	359	17.2% without mesh 1% with mesh	DLIs & Colostomies	n/a
Lorenz (Lorenz et al., 2019)	2019	Austria	140	15.7%	DLIs & Colostomies	Parastomal hernia, male, BMI, HTN, concomitant ventral hernia
Eklov (Eklöv et al., 2020)	2020	Sweden	216	7.4% clinical and radiographic	DLIs after LAR for rectal cancer	Male. Higher BMI
Mongelard (Mongelard et al., 2020) [36]	2020	Sweden	91	25%	DLIs and Colostomies	None
Bloomfield (Bloomfield et al., 2022)	2021	Australia	171	14.6% clinical and radiographic	DLIs	BMI, ASA 3–4

**Table 2:** Analysis of patient demographics and risk factors at the time of DLI-R

Variable	All *N* = 569 (100%)	(-) ISIH 366 (64.3%)	(+) ISIH 203 (35.7%)	*p*-value
**Age**	54.8 (14.9)	54.2 (15.6)	56.0 (13.6)	0.14
**Female**	289 (50.8%)	181 (49.5%)	108 (53.2%)	0.39
**Race**				0.75
White	478 (84%)	304 (83.1%)	174 (85.7%)	
Black	59 (10.4%)	39 (10.7%)	20 (9.9%)	
Other	19 (3.3%)	13 (3.6%)	6 (3%)	
Unknown	13 (2.3%)	10 (2.7%)	3 (1.5%)	
**Ethnicity**				0.65
Hispanic	16 (2.8%)	12 (3.3%)	4 (1.9%)	
Not Hispanic	548 (96.3%)	351 (96.4%)	197 (96.1%)	
Unknown	5 (0.9%)	3 (0.8%)	2 (1%)	
**BMI**,** kg/m**	27.6 (13–53)	26.1 (22.1–29.1)	30.4 (26-34.1)	< 0.001
Normal: 18–25 kg/m^2^	194 (34.1%)	162 (44.5%)	32 (15.6%)	
Underweight: <18 kg/m^2^	14 (2.5%)	11 (3%)	3 (1.5%)	
Overweight: 25–30 kg/m^2^	176 (30.9%)	112 (30.8%)	64 (31.2%)	
Obese ≥ 30 kg/m^2^	185 (32.5%)	81 (22.3%)	104 (50.7%)	
**Smoking Status**				0.82
Active	67 (11.8%)	42 (11.5%)	25 (12.3%)	
Former	237 (41.7%)	150 (41%)	87 (42.9%)	
Never	265 (46.6%)	174 (47.5%)	91 (44.8%)	
**Prior Parastomal hernia**	87 (15.3%)	45 (12.3%)	42 (20.7%)	0.008
**Co-Morbidities**				
Any	223 (39.2%)	132 (36.3%)	91 (44.4%)	0.04
Liver Disease	17 (3%)	11 (3%)	6 (3%)	0.97
CAD	69 (12.1%)	44 (12%)	25 (12.3%)	0.92
CHF	41 (7.2%)	26 (7.1%)	15 (7.4%)	0.90
MI	24 (4.2%)	16 (4.4%)	8 (3.9%)	0.81
TIA/ Stroke	30 (5.3%)	20 (5.5%)	10 (4.9%)	0.78
PVD	24 (4.2%)	14 (3.8%)	10 (4.9%)	0.53
COPD	56 (9.8%)	33 (9%)	23 (11.3%)	0.37
CKD	57 (10%)	33 (9%)	24 (11.8%)	0.29
DM	105 (18.5%)	60 (16.4%)	45 (22.2%)	0.09
**Time to CT**,** days**	321.0 (97.1)	320.3 (99.2)	322.4 (93.3)	0.80

Other = American Indian, Asian, Alaskan, Pacific Islander, multiracial, refused

The figures represent the mean (SD), median (IQR), or frequency (proportion)

**Table 3:** Analysis of operative factors of DLI-R surgery

	All *N* = 569 (100%)	(-) ISIH 364 (64.3%)	(+) ISIH 203 (35.7%)	*p*-value
**LOS**, days	3.76 (4.1)	3.78 (4.1)	3.74 (4.1)	0.92
**Operative Time**, min	150.5 (54.8)	150.3 (50.8)	150.9 (61.5)	0.91
**EBL**, mL	25.8 (69.8)	25.8 (75.5)	25.9 (57.7)	0.99
**Suture Material for Fascial Closure**				0.01
Polydioxanone (PDS^®^)	323 (56.8%)	220 (60.1%)	103 (50.6%)	
Polyglyconate (Maxon™)	184 (32.3%)	100 (27.3%)	84 (41.4%)	
Polyglactin (Vicryl^®^)	11 (1.9%)	8 (2.2%)	3 (1.5%)	
Polypropylene (Prolene^®^)	18 (3.2%)	16 (4.4%)	2 (1%)	
Other	13 (2.3%)	8 (2.2%)	5 (2.5%)	
Not Mentioned	20 (3.5%)	14 (3.8%)	6 (3%)	
**Fascial Suture Technique**				0.37
Running	120 (21.1%)	71 (18.4%)	49 (24.1%)	
Interrupted (including figure-of-eight)	371 (65.2%)	242 (66.1%)	129 (63.6%)	
Not Mentioned	78 (13.7%)	53 (14.5%)	25 (12.3%)	
**Stoma Closure Technique**				0.85
Local Reversal	529 (93%)	339 (92.7%)	190 (93.6%)	
Included Laparotomy	26 (4.6%)	17 (4.6%)	9 (4.4%)	
Included Laparoscopy	14 (2.5%)	10 (2.7%)	4 (2%)	

The figures represent frequencies (proportions) or means (SD)

**Table 4:** Multivariate Logistic Regression Model of Risk factors for Post- DLI reversal Incisional Hernias

Risk Factor	OR (95% CI)	*p*-value
**BMI**, kg/m^2^		
Normal: 18–25 kg/m^2^	1.0 (reference)	
Underweight: <18 kg/m^2^	1.52 (0.32–5.38)	0.5
Overweight: 25–30 kg/m^2^	3.10 (1.88–5.20)	< 0.001
Obese ≥ 30 kg/m^2^	7.19 (4.35–12.2)	< 0.001
**Any Co-Morbidity**		
None	1.0 (reference)	
Any	1.45 (0.90–2.33)	0.13
**Parastomal Hernia**		
No	1.0 (reference)	
Yes	1.15 (0.69–1.90)	0.6
**Suture Material**		
Polydioxanone (PDS^®^)	1.0 (reference)	
Polyglyconate (Maxon™)	2.12 (1.41–3.19)	< 0.001
Polyglactin (Vicryl^®^)	0.87 (0.17–3.42)	0.8
Polypropylene (Prolene^®^)	0.30 (0.05–1.16)	0.13
Other	1.94 (0.51–6.74)	0.3
Unknown	1.08 (0.35–2.97)	0.9

## Electronic supplementary material

Below is the link to the electronic supplementary material.

Supplementary Material 1

## Data Availability

The data generated during this study are available from the corresponding author upon reasonable request.

## Declarations

### Originality

This study is original and has not been published previously. It was presented as an oral presentation at the American Hernia Society meeting on September 22, 2023 in Austin, TX. Artificial intelligence was not used in this study or in the drafting of the manuscript. Paperpal© was used to edit the final draft for grammar and punctuation.
