# Supplementary material for: The rate of ileostomy site incisional hernias: more common than we think?
Source: Hernia. 2024 Sep 26;28(6):2311–20. doi: 10.1007/s10029-024-03163-0 (PMC11530484; doi:10.1007/s10029-024-03163-0)
Supplement: Supplementary file 1 — Supplementary Material 1 [file 10029_2024_3163_MOESM1_ESM.docx]

**Supplementary Table 1:** Demographic comparison of those excluded vs included in the final analysis

| **Variable** | **Included**  **569 (25.9%)** | **Excluded**  **1627 (74.1%)** | **p-value** |
| --- | --- | --- | --- |
| **Age** | 54.8 (14.9) | 50.6 (16.8) | < 0.001 |
| **Female** | 289 (50.8%) | 805 (49.5%) | 0.74 |
| **Race** |  |  | 0.002 |
| White | 478 (84%) | 1414 (86.9%) |  |
| Black | 59 (10.4%) | 104 (6.4%) |  |
| Other | 19 (3.3%) | 60 (3.7%) |  |
| Unknown | 13 (2.3%) | 17 (1.0%) |  |
| **Ethnicity** |  |  | 0.32 |
| Hispanic | 16 (2.8%) | 29 (1.8%) |  |
| Not Hispanic | 548 (96.3%) | 1547 (95.1%) |  |
| Unknown | 5 (0.9%) | 19 (1.2%) |  |
| **BMI, kg/m** | 27.6 (13–53) | 26.4 (22.1–29.6) | < 0.001 |
| **Smoking Status** |  |  | 0.01 |
| Active | 67 (11.8%) | 204 (12.5%) |  |
| Former | 237 (41.7%) | 561 (34.4%) |  |
| Never | 265 (46.6%) | 830 (51.1%) |  |
| **Co-Morbidities** |  |  |  |
| Any | 223 (39.2%) | 502 (30.9%) | < 0.001 |
| Liver Disease | 17 (3%) | 36 (2.2%) | 0.05 |
| CAD | 69 (12.1%) | 170 (10.4%) | < 0.001 |
| CHF | 41 (7.2%) | 97 (6.0%) | < 0.001 |
| MI | 24 (4.2%) | 40 (2.5%) | 0.03 |
| TIA/ Stroke | 30 (5.3%) | 62 (3.8%) | 0.004 |
| PVD | 24 (4.2%) | 86 (5.3%) | 0.02 |
| COPD | 56 (9.8%) | 103 (6.3%) | < 0.001 |
| CKD | 57 (10%) | 145 (8.9%) | < 0.001 |
| DM | 105 (18.5%) | 193 (11.9%) | < 0.001 |

Other = American Indian, Asian, Alaskan, Pacific Islander, multiracial, refused.

The figures represent the mean (SD), median (IQR), or frequency (proportion).
